# Efficacy of Filter Trocar for Clear Visualization during Laparoscopic Cholecystectomy: A Prospective Randomized Controlled Trial

**DOI:** 10.3390/jpm14020204

**Published:** 2024-02-13

**Authors:** Ho-Chang Chae, Beom-Jin Kim, Yoo Shin Choi, Suk-Won Suh, Seung Eun Lee

**Affiliations:** 1 Samsung Medical Center, Division of Hepatobiliary-Pancreatic Surgery, Department of Surgery, Sungkyunkwan University School of Medicine, Seoul 06351, Republic of Korea; chaehochangc@gmail.com; 2 Department of Surgery, Seoul Song-do Hospital, Seoul 04597, Republic of Korea; kbj5555@caumc.or.kr; 3 Department of Surgery, Chung-Ang University College of Medicine, Seoul 06974, Republic of Korea; yschoi@cau.ac.kr (Y.S.C.); bumboy1@cau.ac.kr (S.-W.S.)

**Keywords:** laparoscopic, view, clear, smoke

## Abstract

Filter trocar designed to eliminate harmful smoke is also regarded as effective for improving surgical visualization. The aim of this study is to evaluate the efficacy of filter trocar in maintaining clear operative view. From 2019 to 2020, 100 patients underwent laparoscopic cholecystectomy and they were randomized to either the control or filter group. The primary end point was a laparoscopic operative view score (1, clear; 2, slightly blurry; 3, completely blurry) during gallbladder dissection from the liver bed when dissection was started (LV1), when dissection was half completed (LV2) and when dissection was completed (LV3). Between the control and filter groups, there were no significant differences in mean LV1 (1.44 vs. 1.40, *p* = 0.234) and LV3 (1.86 vs. 2.01, *p* = 0.880). There was no significant difference in the mean duration of suction after dissection (3.82 s vs. 3.67 s, *p* = 0.097) and the mean number of laparoscope removals from inside to outside the body to clean during gallbladder dissection from the liver bed (0.55 vs. 0.22, *p* = 0.963) or the mean amount of time required to dissect the gallbladder from the liver bed (221.58 s vs. 177.09 s, *p* = 0.253). The study demonstrated that filter trocar is not as effective as expected in the maintenance of clear operative view. Further study is needed to develop devices to improve clear surgical visualization.

## 1. Introduction

Maintaining a clear field of view is paramount in laparoscopic procedures not only for safety by preventing inadvertent injury, but also to improve precision and reduce operative time. Recently, with technological advancement, various energy-based surgical devices, such as monopolar or bipolar electrocautery devices, ultrasonic devices and laser systems, have shortened operation time. However, these devices also produce large amounts of surgical smoke during surgery which contains a mixture of water vapor, ultrafine particles and vaporized materials [1]. Compared to open surgery, confined spaces associated with laparoscopic procedure worsen the surgical view. The smoke generated from use of energy-based surgical devices often interferes with clear visualization of the surgical field in laparoscopic surgery and potentially increases risk of intra-operative complications [2]. The aerosolized particles in smoke can impair clear visualization by sticking to the lens of the laparoscope or by floating in the closed body space. Removing particles from the lens of a laparoscope requires pulling the laparoscope out from the body cavity for cleaning and removing floating particles or smoke involves venting or aspiration. These interventions take time to re-establish the adequate visualization of the surgical field and can impede surgical progress. 

The smoke generated during laparoscopic surgery contains various harmful gases such as benzene, toluene, ethyl benzene styrene and formaldehyde. These are known as volatile organic compounds (VOCs), and they have been reported to cause acute or delayed toxicity and have potential carcinogenic effects on humans [3,4]. When smoke escapes from the abdominal cavity, the risk of inhalation by the surgical team increases and especially during the COVID-19 pandemic, the surgical team has become more aware of the dangers associate with surgical smoke, recognizing it as a potential carrier of harmful particles [5,6,7,8]. Various laparoscopic surgical smoke removal solutions have been developed, including filters and suction devices [9,10,11,12]. Filters made of various materials and thicknesses of fibers that can block these harmful gases are developed and have been used [9,10,11]. Several reports have demonstrated that the use of a filter in laparoscopic surgery significantly removes and reduces these harmful gases [9,10,11]. Generally, these laparoscopic filters are regarded as effective instruments for improving surgical visualization via smoke elimination and actually reduce the overall operative time. However, to our knowledge, no published studies have evaluated the efficacy of laparoscopic filters for maintaining a clear surgical view.

Laparoscopic cholecystectomy is among the most common laparoscopic procedures in the world. In laparoscopic cholecystectomy, during gallbladder dissection from the liver bed, the tissue generates a lot of smoke and this is the stage of the procedure most likely to be associated with surgical smoke disrupting a clear laparoscopic view. In general, we can remove the smoke using suction or opening the vent valve of the trocar, but these interventions rapidly decrease intra-abdominal pressure requiring the surgeon to wait until the CO_2_ is fully insufflated and the proper level of pressure is achieved. Moreover, moisture is usually collected that smudges the laparoscope lens, interfering with visibility, requiring repeated camera withdrawal to clean the lens and often adding to the operative time. However, when a filter is connected to the trocar, the intra-abdominal pressure does not decrease so fast and the surgeon does not need to wait for CO_2_ insufflation. Therefore, this study aimed to evaluate the efficacy of the filter trocar for maintaining a clear operative view in laparoscopic surgery.

## 2. Materials and Methods

A single-center prospective randomized controlled trial was conducted from September 2019 to August 2020 in Chung-Ang University Hospital. The study protocol was approved by the institutional review board of Chung-Ang University Hospital (IRB No. 1961-003-375) and was registered with Clinical Research Information Service (CRiS) of the Republic of Korea (identifier:KCT0004435, 14 November 2019). The study was reported in accordance with the rigor of the CONSORT guideline, and all experimental conditions conformed to the Declaration of Helsinki. All patients signed a written informed consent form before enrolment in the study. The filter used in this study was the SMOKE FILTER^TM^ (Endovision, Seoul, Republic of Korea) as a complex filter, which has multiple layers of special filters, including activated carbon fiber, ultra-low particulate air (ULPA) and anti-viral filters. The filter was connected to the channel for the gas vent of the trocar.

### 2.1. Inclusion and Exclusion Criteria 

Patients scheduled for elective laparoscopic cholecystectomy for gallstone disease or gallbladder polyp removal between 18 and 85 years of age with the capacity to provide informed consent were included. Exclusion criteria were those who were less than 18 years old; lactating or pregnant; had evidence of previous extensive abdominal surgery; or current enrollment in another drug or device study. 

### 2.2. Sample Size Calculation 

The sample size was calculated using the difference of time spent waiting or suctioning smoke for clear visualization. There has been no similar studies to guide the calculation, but a difference of 2 min was considered to be clinically significant. To detect a difference of 2 min as statistically significant, more than 45 patients would be required in each study arm based on the following assumption: α = 0.05, power = 0.80, within-group standard deviation = 2 min and ratio of control to experimental patients = 1. Assuming a dropout rate of 10%, a total of 100 participants was required, with 50 participants per group.

### 2.3. Randomization

A block randomization method was used. Enrolled patients were randomized at the time of their operation to either the trocar with filter group or trocar without filter group (control group) in a 1:1 ratio using a randomization table created before the surgery. This was undertaken by an independent surgical research nurse. 

### 2.4. Surgical Procedure

All surgical procedures were performed by two surgeons in the same manner as the three-trocar technique. These surgeons had each performed more than 1000 laparoscopic cholecystectomies; this acted to standardize the variability of surgical skills. Pneumoperitoneum was maintained between 12 and14 mmHg. Under the xyphoid process, a 5 mm trocar was inserted for the operator’s right-hand access. Along the mid-clavicular line, below the costal margin, another 5 mm trocar with or without a filter was inserted for the operator’s left-hand access. Gallbladder dissection from the liver bed was carried out with a monopolar cauterizer. In the control group, the channel for a gas vent in a trocar was opened during gallbladder dissection from the liver bed. In the filter group, the connected channel to filter was opened during the whole procedure. Operative procedures were video recorded onto a standard high-definition recording device and subsequently transferred to individual high-definition memory cards, labeled with the participant’s identification number. The videos began with the start of monopolar hook dissection of the gallbladder from the liver bed, and they ended when the gallbladder was completely free. The suction of smoke using a suction device was performed only once after gallbladder removal. 

### 2.5. Endpoints

**Figure 1:**
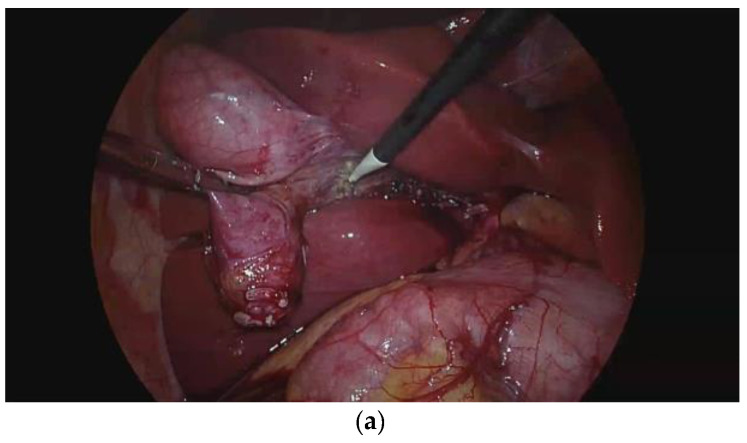

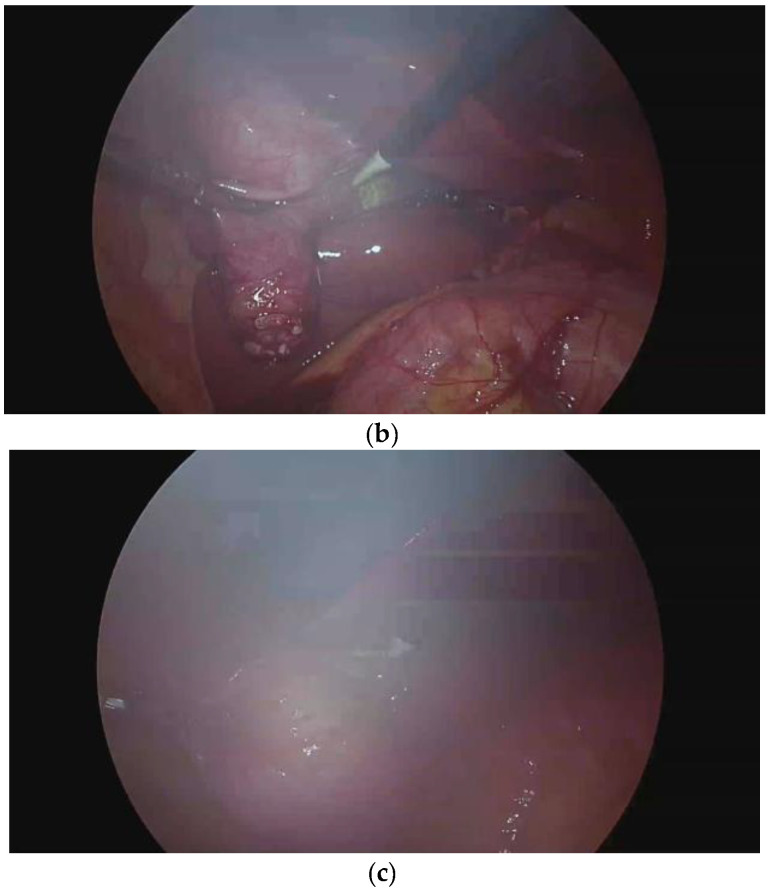
Laparoscopic view score. (**a**) Score 1: the laparoscopic view is clear. (**b**) Score 2: the laparoscopic view is slightly blurry. (**c**) Score 3: the laparoscopic view is completely blurry.

The primary endpoint of this study was the laparoscopic view score during dissection of the gallbladder from the liver bed. The scoring system consisted of a 3-point scale according to blurriness, at three different moments. A score of 1 was applied when the laparoscopic view was clear, a score of 2 was applied when the laparoscopic view was slightly blurry and a score of 3 was applied when laparoscopic view was completely blurry (Figure 1). The laparoscopic view (LV) was also scored at different moments. LV1 was at the beginning of dissection, LV2 was when dissection was half completed and LV3 was when dissection was completed. Two surgeons who did not participate in the surgery reviewed the surgery videos and determined the scores.

The secondary endpoints were the duration of suction after gallbladder removal to maintain a clear surgical view, the number of laparoscope removals from inside to outside of body for cleaning during gallbladder dissection from the liver bed and the total time required for gallbladder dissection from the liver bed. Two surgeons who did not participate in the surgery reviewed the surgery videos and assessed these endpoints. 

### 2.6. Data Collection and Statistical Analysis

Patient demographic and clinical data (age, gender, body mass index, history of prior abdominal surgery) were collected at the time of enrollment. Procedural data such as American Society of Anesthesiology Classification (ASA), estimated blood loss (EBL) and intra-operative complications were collected at the time of the procedure. Statistical analysis was performed with two-sided χ^2^ test using SPSS Statistics for Windows, version 24.0 (IBM Corp., Armonk, NY, USA). All analyses were performed according to the intention-to-treat principle. Data are presented as mean ± standard deviation (SD) or median. Student’s *t* test or the Mann–Whitney test was used to compare means or medians, and the χ^2^ or Fisher’s exact test was used to compare the frequency distributions between the categorical variables, as appropriate. A *p*-value < 0.05 was considered statistically significant. 

## 3. Results

**Figure 2:**
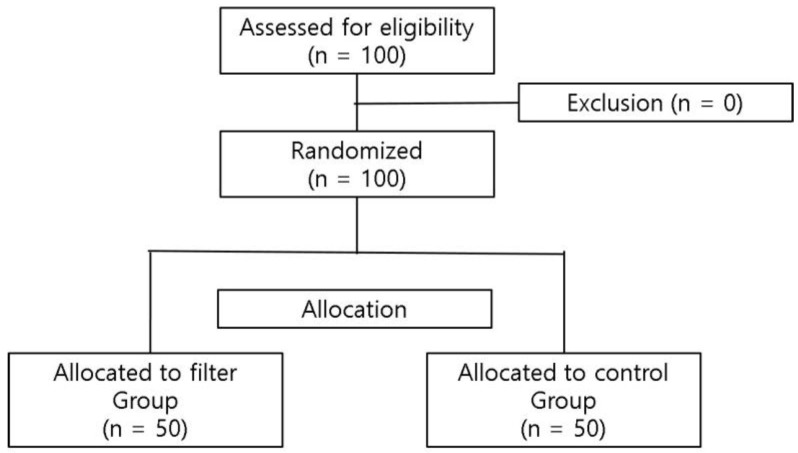
CONSORT patient inclusion flow chart. This study was registered with Clinical Research Information Service (#KCT0004435).

One hundred patients were assessed for eligibility. No patients were excluded. Therefore, 100 patients were randomized, with 50 patients allocated to the filter group and the other 50 allocated to the control group (Figure 2). All participants completed the intervention and there was no exclusion from analysis.

### 3.1. Clinicopathologic Characteristics

There were no significant differences between the filter group and the control group in age, sex, comorbidities, previous operative history, body mass index, total operation time, histopathologic results and postoperative complications (Table 1). There was one postoperative complication (incisional hernia through the umbilical port site in a patient in the filter group).

### 3.2. Primary Endpoint

In the filter group, the laparoscopic operative view was significantly blurrier when dissection was half completed (LV2; 1.94 vs. 1.88, *p* = 0.027). However, there were no significant intergroup differences in LV1 score (1.4 vs. 1.44, *p* = 0.234), LV3 score (2.01 vs. 1.86, *p* = 0.880) and overall LV score (1.65 vs. 1.56, *p* = 0.397) (Table 2).

### 3.3. Secondary Endpoint

There were no significant intergroup differences in the duration of suction after gallbladder removal to maintain a clear surgical view (3.82 s vs. 3.67 s, *p* = 0.097), the number of laparoscope removals from inside to outside of the body for cleaning during gallbladder dissection from the liver bed (0.55 vs. 0.22, *p* = 0.963) and the total time required for gallbladder dissecting from the liver bed (221.58 s vs. 177.09 s, *p* = 0.253) (Table 3).

## 4. Discussion

Ensuring surgical field visualization is important for safe surgery. Compared with open surgery, laparoscopic surgery is associated with more difficulties with maintaining surgical field visualization due to uncomfortable laparoscopic tools and limited peritoneal space [2]. Furthermore, with technological advancements, newly developed energy-based surgical instruments are widely used and these produce large quantities of smoke [1]. This smoke disturbs the operator’s view of the surgical field, interferes with proper dissection, increases operating time and potentially increases the risk of inadvertent intraoperative organ injury. Recently, the World Society of Emergency Surgery (WSES) expert panel has suggested laparoscopy as the first approach for stable patients undergoing emergency abdominal surgery for general surgery emergencies and abdominal trauma [13]. During emergency laparoscopy, tissue edema or inflammation can make more smoke or evaporated particles often causing an obscure surgical view. Therefore, intraoperative smoke removal is important to facilitate safe laparoscopic procedures during both scheduled and emergency operations and now is the right time to consider and develop effective methods. Although filters for trocar were originally developed for the removal of harmful gases generated during laparoscopic surgery, they are generally regarded to improve surgical visualization via smoke removal. However, the effect of filter trocar to remove and enhance laparoscopic visibility has not been evaluated and to our knowledge, this was the first randomized controlled study to evaluate the efficacy of the filter trocar for maintaining a clear operative view.

For smoke removal from the peritoneal space during laparoscopic surgery, opening of the vent valve in the trocar or suctioning the smoke using a suction device are the most commonly used methods. However, opening of the vent valve in the trocar or suctioning the smoke rapidly deflates the required pneumoperitoneum, making the abdominal cavity smaller and narrowing the surgical field of view. To recover sufficient intra-abdominal pressure and clear visualization, surgeons must wait for several seconds. If these steps are applied several times, it becomes time consuming and impedes operative efficiency. However, when a filter is connected to the trocar, since the intra-abdominal pressure is not decreasing as fast and the surgeon need not wait for CO_2_ insufflation, we hypothesized that a filter is effective for removing smoke and maintaining a clear surgical view. This is the first study to evaluate the potential advantage of filtered trocar for a clear surgical view by removing smoke and surgical plumes. We found that the filter did not improve surgical visualization during laparoscopic surgery and did not decrease the frequency or time of suctioning of smoke and the total time taken to dissect the gallbladder from the liver bed. Although recently developed smoke evacuation systems are theoretically considered effective for maintaining a surgical view during laparoscopic surgery by providing stable pneumoperitoneum and constant smoke evacuation, there has been no comparative study in terms of clear vision [14,15,16,17]. The theoretical advantage of the smoke evacuation system is that this system responds immediately to the slightest changes in intra-abdominal pressure, maintaining a stable-pressure pneumoperitoneum and continuous smoke evacuation even under difficult surgical conditions and constant suction, ensuring adequate visibility [14,15,16,17]. When comparing smoke evacuation systems to the filter trocar in the present study, the latter presents a list of benefits including acceptable price, no need for external system and complicated set-up and no requirement for additional space.

Although filter use was not so effective in maintaining surgical visualization, keeping the trocar open without a filter during surgery is associated with safety problems for medical staff because the smoke generated by using energy-based devices during surgery contains various harmful substances including carcinogens such as toluene, ethyl benzene and butadiene [3,4,18]. Baggish et al. confirmed a relationship between inhalation of unfiltered surgical smoke and changes in lung conditions including alveolar congestion and emphysema in a rat model [19,20] and several reports demonstrated that surgical smoke has potentially dangerous effects on medical staff [5,6,7,8,21]. The US Centers for Disease Control recommended the installation of smoke removal devices in the operating room and the use of N95 masks by medical staff during surgery [22]. In this regard, the use of filter trocar during laparoscopic surgery should be considered because the effect of filter trocar in term of removal of surgical smoke has been proven [9,10,11] and especially in Korea, the price of filter trocar is the same as regular trocar.

There are several limitations in this study. Firstly, since laparoscopic cholecystectomy was performed by an experienced surgeon, the total duration of gallbladder dissection from the liver bed was only within 4 min (221 s in the filter group and 177 s in the control group). Therefore, the difference in overall time and the time required for smoke suction between the two groups was likely to be minimal. For less-experienced staff, to achieve clear surgical visualization, removal of smoke may be more time consuming and a statistical significance could be achieved. It is necessary to evaluate the outcome of filter trocar on complex procedures with longer operating time and higher smoke production. Secondly, our scoring system for clear visualization was a subjective categorical measurement and dependent on the experimenters. However, to overcome this limitation, two surgeons evaluated the clarity of visualization.

Recently, a substantial number of computer-based visualization techniques which help in modelling different smoke components and lead to smoke-free images have been proposed to restore visibility in the context of hazy surgical fields [23,24,25]. However, they are undergoing validation studies, and it is expected to take some time before such technology is commercialized.

## 5. Conclusions

The present study demonstrated that the laparoscopic operative view during dissection of the gallbladder from the liver bed was not clearer in the filter group than the in-control group. Therefore, a filter for trocar use does not represent an improvement over a lack of use to maintain a clear operative view in laparoscopic surgery. However, we believe that further research and development should enhance the filtering efficiency and further study is needed to develop or identify the new devices or methods for smoke clearance and improvement of clear surgical visualization.

## Figures and Tables

**Table 1:** Clinicopathologic characteristics of the patients.

	Filter Group (n = 50)	Control Group (n = 50)	*p*-Value
Age (years)	47.7	50.5	0.300
Sex, M:F	21:29	25:25	0.433
Comorbidity	15 (29.4%)	12 (24.5%)	0.579
DM	7 (13.7%)	4 (8.2%)	0.374
Hepatitis	2 (3.9%)	4 (8.2%)	0.372
Hypertension	7 (13.7%)	6 (12.2%)	0.826
Previous op hx.	12 (23.5%)	15 (30.6%)	
BMI	25.2	24.7	0.491
Operation time (min)	43.2	41.6	0.560
Pathologic result			
Chronic cholecystitis	42 (82.4%)	38 (79.2%)	0.338
GB polyp	9 (17.6%)	8 (16.7%)	0.230
GB cancer	0 (0.0%)	2 (4.2%)	0.535
Postoperative complication	1 (2%) *	0 (0%)	0.325

*; incisional hernia through umbilical port site.

**Table 2:** Primary endpoints: the laparoscopic view scores.

	Filter Group (n = 50)	Control Group (n = 50)	*p*-Value
LV* 1 score	1.4	1.44	0.234
LV2 score	1.94	1.88	0.027
LV3 score	2.01	1.86	0.880
LV average score	1.65	1.56	0.397

LV*; laparoscopic view.

**Table 3:** Secondary endpoints: duration of suction after gallbladder removal to maintain a clear surgical view, numbers of laparoscope removals from inside to outside of body for lens cleaning during gallbladder dissection from the liver bed and total time required for gallbladder dissection from the liver bed.

	Filter Group (n = 50)	Control Group (n = 50)	*p*-Value
Duration of suction after gallbladder removal (s)	3.82	3.67	0.097
Number of laparoscope removals (times)	0.55	0.22	0.963
Total time required for gallbladder dissection (s)	221.58	177.09	0.253

## Data Availability

Data related to the manuscript are available upon request to corresponding author.
